# A scoping review of school-based oral health interventions among adolescents in Nigeria

**DOI:** 10.3389/froh.2025.1577753

**Published:** 2025-07-29

**Authors:** Abel Nnamdi Chukwuemeka, Richard Oveh, Anita Dabar, Omorinola Afolabi, Saheed Ademola Ibraheem, Folahanmi Tomiwa Akinsolu, Abideen Olurotimi Salako, Omolola Titilayo Alade, Foluso Owotade, George Uchenna Eleje, Oliver Ezechi, Joanne Lusher, Moréniké Oluwátóyìn Foláyan

**Affiliations:** ^1^ Oral Health Initiative, Nigerian Institute of Medical Research, Yaba, Lagos, Nigeria; ^2^ Department of Public Health, Lead City University, Ibadan, Oyo, Nigeria; ^3^ Department of Planning, Research, Monitoring and Evaluation, Lagos State Health Management Agency, Alausa, Lagos, Nigeria; ^4^ Department of Information and Communication Technology, University of Delta, Agbor, Delta, Nigeria; ^5^ Department of Social Development, Moshood Abiola Polytechnic, Abeokuta, Ogun, Nigeria; ^6^ Scientific and Industrial Research Department, National Research Institute for Chemical Technology, Zaria, Kaduna, Nigeria; ^7^ Department of Clinical Sciences, Nigerian Institute of Medical Research, Yaba, Lagos, Nigeria; ^8^ Department of Community and Preventive Dentistry, Obafemi Awolowo University, Ile-Ife, Osun, Nigeria; ^9^ Department of Oral Medicine, Obafemi Awolowo University, Ile-Ife, Osun, Nigeria; ^10^ Effective Care Research Unit, Department of Obstetrics and Gynaecology, Nnamdi Azikiwe University, Awka, Nigeria; ^11^ Provost’s Group, Regent’s University London, London, United Kingdom; ^12^ Department of Child Dental Health, Obafemi Awolowo University, Ile-Ife, Osun, Nigeria

**Keywords:** dental health, tooth brushing frequency, fluoridated toothpaste, cariogenic diet, dental service utilization, young people, youth, telehealth

## Abstract

**Background:**

This scoping review aimed to map school-based oral health interventions for adolescents in Nigeria and to identify gaps that can be addressed to promote optimal oral health in the population.

**Methods:**

This scoping review is registered with the Open Science Framework (OSF) Registries (https://doi.org/10.17605/OSF.IO/CMRV4). An electronic search of PubMed, Scopus, Web of Science, African Journals Online, and Google Scholar for papers focused on oral health interventions implemented in schools in Nigeria, involving adolescents, and published in English between January 2000 and July 2024 was retrieved. Information extracted included study characteristics (author, year, study design), study population characteristics, type of interventions, focus, and key findings.

**Results:**

The search yielded 392 results, with five studies meeting the eligibility criteria. All studies focused on oral health education, and education was delivered verbally. Four studies assessed the effect of education on oral hygiene practices, three assessed the effect on the consumption of refined carbohydrates, two measured dental service utilization as an outcome, and one assessed smoking cessation. One study showed that peer- and teacher-led models demonstrated comparable effectiveness, making them viable in resource-limited settings.

**Conclusion:**

This scoping review found that school-based oral health interventions effectively improved oral hygiene among adolescent populations in Nigeria, though the few studies skewed to southern Nigeria make it difficult to generalize findings to Nigeria.

**Review registration:**

OSF, https://doi.org/10.17605/OSF.IO/CMRV4.

## Introduction

Adolescents are one of the most important demographic groups in terms of oral health due to their susceptibility to various oral health issues influenced by biological, social, and environmental factors (1). Poor oral health is associated with multiple complications, including pain and discomfort, tooth loss, reduced oral functioning, deformity, school absence, and mortality from oral cancer (2). Oral health issues among adolescents have a long-term impact on general health and well-being. The adverse impact of oral health on general health includes reduced quality of life and risk of diabetes and coronary artery disease (3, 4). Predispositions to poor oral health among adolescents include poor dietary habits, eating disorders, use of alcohol and drugs, teen pregnancy, and various other social and psychological determinants of health (5).

Furthermore, oral diseases are a major public health concern for adolescents in Africa. For example, in 2019, nearly one-third (31.0%) of adolescents across the African continent had untreated caries in permanent teeth (6). In the same period, the prevalence of untreated caries in permanent teeth among 10–19-year-old adolescents in the World Health Organization African Region was 29.8%, and the prevalence of periodontal diseases was 1.7% (6).

The incidence of oral diseases among adolescents in West Africa has also been increasing over the past 30 years, with Nigeria having the highest prevalence of oral diseases (7). Worldwide, about half of all adolescents require dental healthcare, and 34% have unmet dental healthcare needs (8). These indices reflect unmet dental needs among adolescents in West Africa and Nigeria, a global problem. In Nigeria, 15%–58% of adolescents over the age of 15 years present with periodontal disease, which significantly affects the quality of life (9). The pooled caries prevalence among adolescents is 23%, and the adjusted prevalence ratio of having caries increased by 18% for every additional age year (10, 11). In addition, only 8.7% of adolescents reported brushing their teeth twice daily, 16.1% used dental floss daily, 1.1% used dental services in the previous 12 months, and 36.1% consumed refined carbohydrates between meals less than once daily (11). Furthermore, poor oral health status among adolescents is linked to poor mental and sexual health (12). The establishment of health insurance initiatives has not significantly reduced the prevalence of oral disease or access to oral health among adolescents, thus indicating that further upstream policy changes may be required (13).

Moreover, preventive oral health education programs (education on oral hygiene practices, dietary guidance, awareness of oral health risks, encouraging regular dental visits and behavioral interventions addressing habits like smoking, nail-biting, or using teeth as tools, which can harm oral health) and community-based initiatives can promote good oral health (14, 15). Oral health interventions among children in primary schools using skills-based education and improving access to oral health services reduce the burden of oral disease (16). In Nigeria, a four-year oral health intervention among children in primary schools using education increased the uptake of fluoride-containing toothpaste, tooth brushing frequency, and sugar-containing snacks consumption (17). Educational interventions can significantly improve students’ knowledge, attitude, and behavior compared to those receiving usual care (18).

Oral health interventions can enhance access to dental care, strengthen oral health systems, and reduce oral health disparities among populations. It can enhance access to dental care among adolescents (19). Policy reforms, fluoridation programs, national health policies, and initiatives to regulate sugary snack marketing and tobacco control policies can also foster optimal oral health (20). School-based oral health education programs represent one of the most widely used interventions that can provide adolescents with knowledge and skills to maintain good oral hygiene practices, including oral hygiene practices, good dietary habits, and tobacco avoidance (21). These programs often incorporate interactive activities, peer education, and collaboration with dental professionals to engage adolescents effectively (22, 23).

School-based oral health interventions have emerged as a promising strategy for addressing adolescents’ oral health needs in Africa, where access to dental care remains limited (17). School-based programs can potentially improve oral health self-care practices among children and, if well-designed and implemented, can improve the oral health outcomes of adolescents in Nigeria (24). This underscores the need for a comprehensive review of the existing evidence on school-based oral health interventions for adolescents in Nigeria.

This scoping review aimed to systematically map and analyze the characteristics of the interventions, components of oral health interventions, variations in how educations are conducted, and to identify the gaps in adolescent-targeted school-based oral health interventions in Nigeria.

## Methods

This scoping review process utilized three frameworks: Arksey and O'Malley Framework, Population Concept Context (PCC) Framework, and PRISMA-ScR guidelines (25, 26). It was registered with Open Science Framework (OSF) Registries (https://doi.org/10.17605/OSF.IO/CMRV4). The Arksey and O'Malley framework consists of five main steps: formulating the research questions, identifying relevant studies, selecting the studies, organizing the data, and compiling, summarizing, and presenting the findings. The target population for this scoping review were adolescents in Nigeria. The review assessed the various types of oral health interventions implemented among the individual, the strategies adopted and the implementation processes. The geographical context for the implementation of these interventions was considered in the assessment.

### Research question

To guide the development of the questions in this scoping review, we used the following PCC (Population, Concept, and Context) strategy: Population: adolescents in Nigeria; Concept: oral health interventions; Context: school. The research questions of the scoping review were: What school-based oral health interventions are available for adolescents in Nigeria? What gaps have been identified to address this population's need for optimal oral health?.

The review's objectives were: (1) to outline the characteristics of school-based oral health interventions targeting adolescents in Nigeria. (2) To examine the specific components of these interventions targeting adolescents in Nigerian schools. (3) To investigate the differences in school-based oral health interventions for adolescents across Nigeria. (4) To identify gaps in implementing these interventions among Nigerian adolescents.

### Identification of relevant studies

A search strategy was developed to identify relevant studies. The search was conducted in PubMed, Scopus, Web of Science, African Journal Online, and Google Scholar using the prespecified search terms. Search terms were identified within titles, abstracts, or keywords. Mapping terms to subject headings (i.e., MeSH terms) was used within applicable databases to increase the efficiency and precision of the search. The search strategies included phrases like “school-based oral health interventions,” “adolescents,” and “Nigeria.” The search strategies used for each database are detailed in Supplementary Appendix 1.

### Eligibility criteria

Studies included in the scoping review focused on oral health interventions implemented in schools in Nigeria that included adolescents 10–19 years old (+2 years). Only complete studies were conducted between January 2000 and July 2024, a critical period that marked the beginning of global and national commitments to improve adolescent health under the Millennium Development Goals and the Sustainable Development Goals. This timeframe allows for studies to be included during these critical policy and programmatic evolution phases. Studies with data that could be independently reported for adolescents within the age range of the study were included in this review. Reviews, editorials, and opinion pieces not addressing study objectives were excluded.

### Study selection process

Articles identified through the search strategy were first organized in EndNote. The records were then imported into Rayyan, where duplicates were removed. Three independent reviewers (AC, RO, and AD) carefully screened the titles and abstracts to identify studies that met the inclusion criteria.

Two reviewers (AC and MOF) retrieved full-text articles for studies deemed potentially relevant. Institutional library resources, online databases, and journal websites were accessed to obtain the full texts. When articles were not immediately accessible, reviewers contacted corresponding authors via email to request copies, searched alternative repositories and conducted citation tracking and cross-referencing to locate alternate sources. Articles that remained inaccessible were documented, and their unavailability was acknowledged as a limitation of the review. Throughout the process, any discrepancies in eligibility assessment were resolved through discussions and consensus among the reviewers, ensuring methodological rigor and consistency.

### Inter-rater reliability

To ensure the reliability of the screening process, inter-rater agreement among reviewers was assessed using Cohen's Kappa statistic for both the title and abstract screening and the full-text screening phases. The average Kappa value was 0.98. For full-text screening, two reviewers independently screened the full-text articles. The inclusion and exclusion criteria were again applied to determine eligibility. The Cohen's Kappa coefficient for the full-text screening was 0.93, with a 98.7% agreement. The high Kappa values signify substantial to almost perfect agreement (27).

### Data charting

Investigators developed a standardized data extraction form for data from included studies. Key information extracted included study characteristics (author details, year of publication, study design), population characteristics, intervention details, outcomes measured, and key findings related to school-based oral health interventions among adolescents in Nigeria.

### Collating, summarizing, and reporting results

Characteristics (evidence source data and population characteristics) and data relating to the review objectives (intervention characteristics, types of Intervention, key outcomes of interventions, and intervention implementation gaps) were collated and presented in a table, and a narrative summary was provided.

In addition, a qualitative analysis of the was findings of the studies was conducted using inductive coding to identify key themes and concepts that capture the main ideas emanating from the study findings and identify gaps or areas needing further research. The coding was developed through an iterative process of reviewing the key study outcomes for patterns and meanings, identifying patterns and relationships between codes, and developing coherent themes that reflect the key findings. The developed themes were shared with team members to validate interpretations. The findings was used to compliment the outcomes of the analysis conducted using ChatGPI 3.5.

## Results

**Figure 1:**
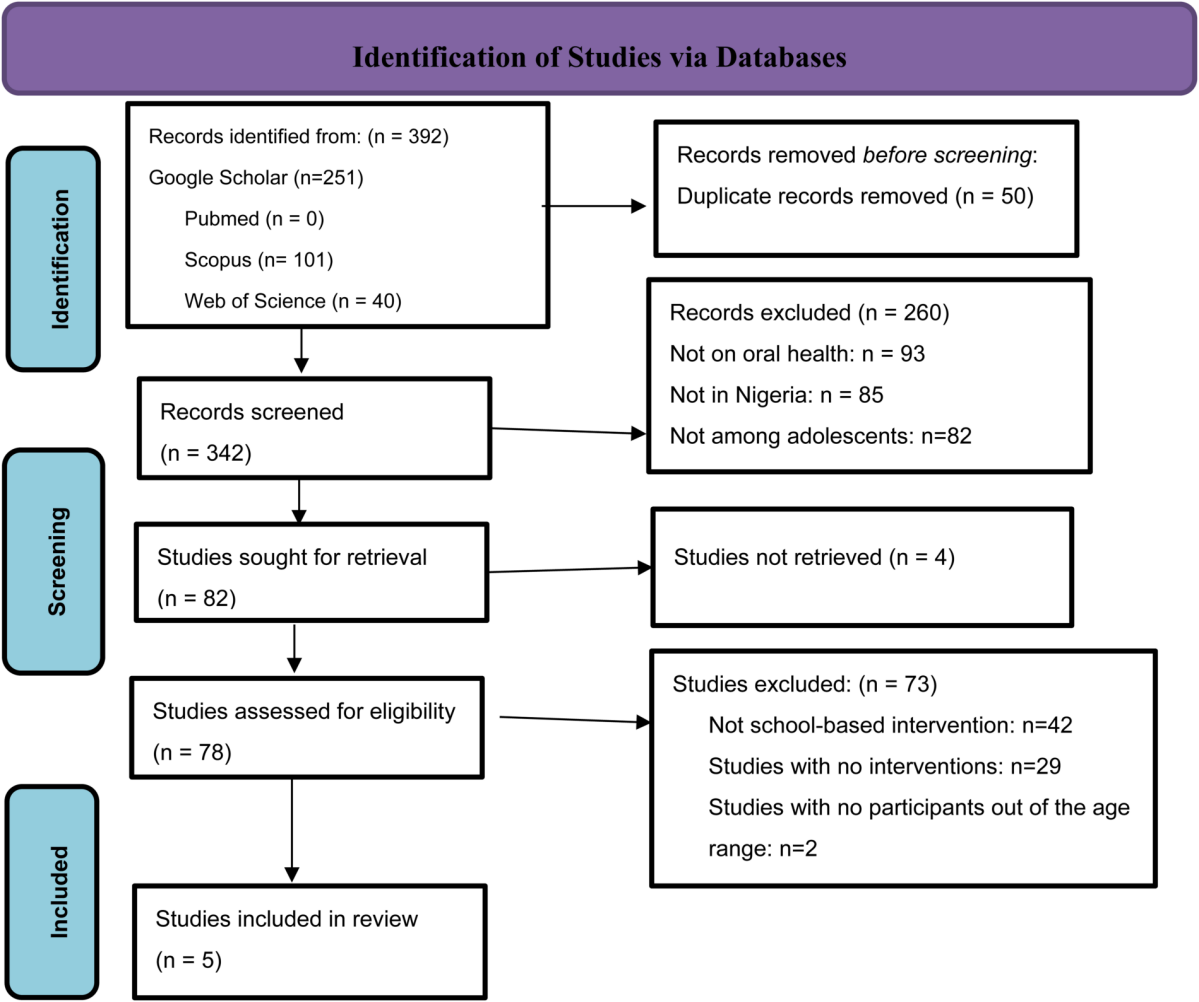
Literature included in the scoping review based on PRISMA 2020 protocol (28).

The initial search yielded 392 records, as shown in Figure 1. Once these records were compiled, 50 duplicate records were removed before screening, leaving 342 records for further review. In the title and abstract screening stage, 260 records were excluded for the following reasons: the studies were not on oral health (*n* = 93), the population were not adolescents (*n* = 82), and not conducted in Nigeria (*n* = 85). A total of 82 studies were sought for retrieval, of which four could not be retrieved. In the full-text screening phase, 73 studies were excluded, with three reasons being that the studies were not focused on a school-based intervention (*n* = 42), no interventions were reported (*n* = 29), and the age of study participants was outside the age range for the current study (*n* = 2). Overall, five (28–32) studies were included in the review.

### Population characteristics

Table 1 shows that the studies were published between 2013 and 2023. The distribution of these studies shows a consistent effort to address oral health issues among adolescents over ten years, with interventions taking place in two of the six geopolitical zones in Nigeria: South-East and South-West geopolitical zones of Nigeria. The study population characteristics varied but primarily targeted adolescents aged between 8 and 21. Three studies were conducted in the South-West region (29, 31, 32), and two were conducted in the South-East (28, 30). The total sample for the included studies was 4073 (1,904 males and 2,169 females), with sample sizes ranging significantly from 1,200 (29) to 1,800 students (31). The included studies recruited participants from primary schools (28, 29, 32) and secondary schools (30, 32).

**Table 1:** Characteristics of the studies included in the scoping review.

S. no	First author	Year of study	City region	Study design	Study objective	Sample size (Male, Female)	Age range	School setting
1	Olubunmi Olusola Bankole (29)	2013	Ibadan South-West	Cohort study	To evaluate the effectiveness of a dental health education video in the Yoruba language for children aged 11–12 years from lower socioeconomic backgrounds in Ibadan, Nigeria.	120 (59, 61)	11–12 years	Three public primary schools in Idikan, Ibadan
2	Nneka Onyejaka (28)	2016	Enugu South-East	Cohort study	To evaluate how school-based education and referral programs influence the utilization of oral healthcare services among primary school children aged 8–11 years.	1,406 (672, 734)	8–11 years	30 primary schools in three LGAs
3	Augustine Ikponmwosa Edomwonyi (32)	2020	Lagos South-West	Quasi-experimental study	To assess the effectiveness of teachers and dentists as agents of oral hygiene education on pupils’ oral health knowledge and practices.	400 (172, 228)	9–14 years	Four public secondary schools in rural and urban Lagos
4	Folake Barakat Lawal (31)	2021	Ibadan South-West	Cluster randomized control trial	To compare peer-led, dentist-led, and teacher-led oral health promotion strategies in schools	1,800 (886, 914)	14–18 years	36 schools in four of the five LGA in Ibadan
5	Henry Uyi Igbinedion (30)	2023	Abakaliki South-East	Quasi-experimental study	To determine the effects of an oral health education package on the knowledge, attitude, and behavior of secondary school students in the urban area of Abakaliki, Nigeria	347 (115, 232)	13–21 years	2 secondary schools in urban Abakaliki

### Study designs

The studies employed a range of research designs, with one study using clustered randomized controlled trials (31), two adopting quasi-experimental designs (30, 32), and two using a cohort design (28, 29). The randomized controlled trial compared dentist-led, teacher-led, and peer-led education (31). One quasi-experimental study compared teacher-led intervention schools and dentist-led Intervention (32).

### Types and tools used for oral health interventions

Table 2 shows that all studies focused on oral health education as the primary Intervention, though the delivery methods varied. All studies conducted verbal education. In addition, one study also used video as an intervention tool (29).

**Table 2:** Details on the oral health interventions conducted by the studies included in the scoping review.

S. no	First author	Type of intervention	Details of intervention	Targeted oral health behavior	Duration of intervention	Point of evaluation of the effect of intervention	Study outcome
1	Olubunmi Olusola Bankole (29)	Oral health education, free toothbrush and toothpaste, scaling and polishing	Three intervention groups: a 20-min video education group, a 20-min verbal education group, and a control group that received no dental health education	Oral hygiene status	One day	6 weeks after the Intervention	Video and verbal education led to improvement in oral hygiene scores when compared to control. The improvement was higher with video education.
2	Nneka Onyejaka (28)	Oral health education and referral	Oral health education and referral of all students for use of preventive and curative dental services	Dental service utilization	One day	12 months after the Intervention	Combining oral health education and referral letters increased dental service utilization for preventive care. Children with low socioeconomic status had lower odds of using dental service.
3	Augustine Ikponmwosa Edomwonyi (32)	Oral health education	Lectures and educational activities conducted by (i) dentists and (ii) teachers for 30 min twice a week for four weeks	Oral health knowledge, attitude, practices (frequency of tooth brushing, fluoride toothpaste use, cariogenic diet, dental service utilization).	Four weeks	6 months after the Intervention	There was a significant improvement in knowledge scores with teachers compared to dentists, although attitude scores were better with dentists than teachers. Better oral health practices observed with dentist than with teachers.
4	Folake Barakat Lawal (31)	Oral health education	Oral health education conducted by a peer (Group I), dentist (Group II) or teacher (Group III)	Frequency, timing and technique of toothbrushing, smoking, cariogenic food consumption, oral health status (gingival health, periodontal treatment needs, decayed teeth, oral hygiene) and oral health-related quality of life	12 months–30 min once every two months for six sessions	6 months after the Intervention	The dentist-led oral health promotion strategy was the most effective at improving oral health knowledge, attitude, practices, oral health status and oral health-related quality of life. Peer-led and teacher-led interventions were comparable.
5	Henry Uyi Igbinedion (30)	Oral health education and free toothbrushes	Educational sessions on oral health with emphasis on timing of tooth brushing and proper tooth brushing technique; tooth brushing practice sessions	Knowledge, attitude and behavior on the frequency of toothbrushing, timing of toothbrushing, technique of teeth brushing, and cariogenic drink consumption	6 weeks – three sessions 2 weeks apart.	4 months after the Intervention	The proportion of children with good knowledge of oral diseases and care, good attitudes toward oral health and good practice increased significantly post-intervention. The knowledge of oral diseases was better in female than male students.

People trained to provide interventions were dentists (31, 32), teachers (31, 32), and peers (31). One of the studies compared the effect of peer-led, teacher-led, and dentist-led education on tooth brushing and dental service utilization (31). One study combined education with a referral program to encourage dental service utilization (28).

### Duration of oral health interventions

The duration of interventions in the included studies ranged from short programs lasting one day (28, 29) to longer interventions that spanned twelve months (31). The post-intervention assessment was conducted for one month (30) to twelve months (28) after Intervention.

### Outcomes measured

The primary outcomes measured across the studies included oral health knowledge, behavior change (tooth brushing frequency, timing and techniques, fluoridated toothpaste use, and refined carbohydrates frequency), and dental service utilization. Four of the studies assessed oral hygiene practices, with a specific focus on the frequency (29–32), timing (30), brushing techniques (29, 30–32), use of fluoride toothpaste (32), and consumption of refined carbohydrates (30–32). In addition, two studies measured dental service utilization as an outcome, aiming to assess whether education alone could influence adolescents to seek preventive care (28, 32) and one assessed smoking (31).

Measures of study outcomes include the use of knowledge (30, 31), attitude (30, 31), behavior/practice (30, 31) scores, oral hygiene status (29, 31), gingival health (31), caries experience (31), periodontal treatment needs (31) and oral health quality of life (31). Number of students who visited dental clinics following referrals was calculated (28). Gender disparities in response to the interventions were also examined, focusing on how male and female students reacted differently to the same educational content (30).

### Key findings

All five studies reported a positive impact on adolescents’ oral health knowledge and behavior. Four key themes that emerge on the effectiveness of oral health interventions and the factors influencing their outcomes highlighted differences in strategy effectiveness, socioeconomic disparities, gender-related outcomes, and long-term retention of oral health practices.

First, regarding educational modality effectiveness, one study showed that video education yields stronger outcomes in improving oral hygiene scores than verbal methods (29). Another study showed that although dentist-led strategies out-performed teacher-led ones in changing attitudes and practices, teacher-led approaches were effective for knowledge retention (32). Both teacher- and peer-led methods demonstrated substantial improvements and had comparable outcomes, making them viable alternatives in settings with limited access to dental professionals (31).

Second, one study showed that combining knowledge and action (pairing education with referral letters enhances) improved preventive dental care utilization. However, children with lower socioeconomic status face barriers in utilizing dental services despite Intervention. This underscores the need for targeted interventions (28).

Third, one study showed that knowledge, attitude, and practice shifts could result from oral health interventions, as there were improvements in understanding the causes of gum disease and tooth decay and shifts in attitudes toward preventive dental visits (30). However, knowledge gains were higher in teacher-led interventions, while dentist-led strategies were more effective in changing attitudes and practices (32). Gender differences were also observed, with female students showing greater improvements in knowledge and behaviors compared to males (31).

Finally, one study assessed the comparative effectiveness of peer and teacher-led models of oral health education interventions and found that peer-led and teacher-led interventions were comparable, suggesting that both education models can be equally effective when properly structured (31).

## Discussion

This study is the first to conduct a comprehensive assessment of the effect and impact of interventions on the oral health of adolescents in Nigeria. First, a limited number of intervention studies are available in the literature, which are restricted to just two of the six geopolitical zones in Nigeria and mainly educational interventions. Second, although the interventions addressed oral hygiene practices, use of fluoride toothpaste, refined sugar consumption and dental service utilization, the effect of the interventions were only reported for oral hygiene practices and use of fluoride toothpaste: none reported on the impact of the interventions on refined sugar consumption. Third, video-based and dentist-led interventions may be more effective in achieving impact.

One of the strengths of this study was the strong focus on behavior change, with interventions generally proving successful at improving key oral health behaviors such as tooth brushing frequency. Including measurable outcomes related to behavior and knowledge provided concrete evidence of the interventions’ effectiveness.

A limitation of the study was the variation in study design, sample size, and intervention duration, making it challenging to compare results across studies. Studies that employed short-term interventions may not adequately capture the long-term sustainability of behavior change or the retention of oral health knowledge over time. In addition, the inability to retrieve four of the 82 screened studies may have resulted in the inadvertent exclusion of publications in the final review. Despite these limitations, the current study contributes to new knowledge on the study matter in Nigeria.

The findings from this scoping review of school-based oral health interventions among adolescents in Nigeria broadly align with several trends observed in global and regional studies on adolescent oral health education (33). For instance, studies conducted in Brazil and India, which share socioeconomic and healthcare challenges like Nigeria, have demonstrated that school-based oral health education can improve oral hygiene practices (34, 35). Much like in Nigeria, studies have shown that multimedia tools (videos, songs, and visual aids) have proven effective in engaging young people and fostering sustained behavior change (36). This indicated that using interactive and engaging methods led to better oral health outcomes than traditional didactic education methods (37).

Oral health education can change oral health behavior by following a pathway that integrates both psychological and social factors, as explained by models like the Cognitive Social Health Information Processing (C-SHIP) model (38) and the Capability, Opportunity, and Motivation (COM-B) model (39). According to the C-SHIP model, oral health education helps individuals perceive, encode, and integrate new information into their beliefs about oral health. Once individuals have formed a knowledge base, it influences their **motivation** to act. In the COM-B model, capability (knowledge) and motivation interact to drive behavioral change. Thus, when people understand the consequences of poor oral health—such as tooth decay or gum disease—they become more motivated to adopt preventive practices like brushing twice a day, using fluoride toothpaste, or seeking dental services for check-ups.

Education alone is not enough; individuals need opportunities to act on their new knowledge. According to the COM-B model, opportunity refers to social and environmental factors. Education and practical opportunities—like accessible dental services, referrals, and affordable care—can bridge the gap between knowledge and action. For example, pairing oral health education with referral letters (as observed in the findings) (28) increased dental service utilization. This suggests that creating accessible pathways to care can drive behavior changes.

As individuals adopt new oral health behaviors (such as increased brushing frequency), these actions reinforce their motivation to maintain good habits. Over time, consistent behaviors improve oral health outcomes, which feeds back into increasing their capability and motivation to continue these practices. This dynamic process ensures that once behaviors are adopted, they can be sustained in the long term. Peer-led and teacher-led interventions can be particularly effective in creating social motivation for behavior change. These models allow for disseminating oral health knowledge in a culturally acceptable and familiar context, enhancing the social opportunity for adopting new behaviors. The findings show that both models can be as effective as dentist-led strategies in knowledge retention, emphasizing the role of social networks in reinforcing healthy behaviors.

The findings on gender differences in how oral health education influences behavior are consistent with another study conducted in Japan that found that women have more positive attitudes towards dental visits, higher oral health literacy, and better oral health behaviors than men (40). This finding raises important questions about how health interventions are designed and whether they adequately account for how boys and girls might engage with health education. In addition, this raise concerns that current oral health interventions, if not tailored to address these gender-specific needs, could be less effective for one gender compared to the other. The finding suggests that interventions should consider gender-specific motivations and barriers when designing oral health education programs to maximize effectiveness.

In addition, the findings on disparities in dental service use among lower socioeconomic groups indicate the need for targeted education and opportunity creation. The finding mirrors observations from studies in countries such as South Africa, where structural barriers, such as financial constraints, affect adolescents’ health-seeking behaviors (41). These findings suggest that while school-based education is necessary, it may not be sufficient without addressing broader socioeconomic determinants of health. Tailoring interventions to consider socioeconomic barriers—such as subsidizing care or increasing community outreach—ensures that disadvantaged populations have both the knowledge and the opportunity to access preventive services, closing the gap in health outcomes.

Interventions integrating multimedia tools, such as videos and interactive content, showed better engagement and outcomes. Multimedia tools have a greater impact on oral health for school children due to their ability to engage, simplify complex information, and enhance retention (42). These tools use visual, auditory, and interactive elements to capture attention and facilitate learning (43). They also support personalized and self-paced learning, with content adaptable to different levels and cultural contexts (44). The current study findings align with the broader literature on health education, which emphasizes using culturally relevant materials to enhance knowledge retention (45). The use of culturally appropriate education materials that are appropriate for adolescent education is more likely to change oral health behavior by increasing capability (knowledge and skills) within school-based created opportunities (social and environmental factors), and enhancing motivation (emotional and psychological readiness) to engage in preventive practices. The process is dynamic, as changes in behavior further reinforce capability, motivation, and access, creating a sustained cycle of improvement in oral health outcomes. This of culturally appropriate education materials not only makes the content more relatable but also increases participants’ motivation to engage with the learning material.

Table 3 is a summary of the findings from the study. It outlines strategies for improving school-based oral health through diverse behavioral change models. The table also includes our thoughts on the possible implications of the study findings for the future. Multimedia education uses culturally relevant tools to enhance short-term knowledge retention, while guided tooth brushing programs with rewards foster consistent habits. Peer-led education leverages relatability to moderately improve outcomes, complementing teacher-led education, which achieves sustained behavior changes through curriculum integration. Dentist-led education proves most effective but resource-intensive, whereas referral programs boost dental service use, albeit with socio-economic barriers. Gender-specific tailoring addresses learning differences, showing greater benefits for female students. Collectively, these strategies emphasize resource-sensitive, tailored interventions to optimize oral health outcomes in schools. Our postulations on the implications of the study findings for future interventions need to be explored by further studies.

**Table 3:** Summary of behavioral change models and strategies in school-based oral health interventions.

Model/strategy	Key features	Effectiveness	Implications for future interventions
Multimedia education	Use of culturally relevant videos, songs, and cartoons tailored to local languages and contexts.	Improved oral hygiene knowledge (with videos) and behavior retention over short-term interventions.	Incorporate culturally tailored multimedia tools to enhance engagement and retention, especially in younger age groups.
Guided tooth brushing	Daily supervised brushing at school with fluoride toothpaste provided, often reinforced by rewards.	Increased frequency of brushing and fluoride toothpaste use.	Implement structured daily brushing programs with visual or reward systems to instill consistent habits early in childhood.
Peer-led education	Oral health education sessions led by student peers to encourage relatability and peer influence.	Moderate improvements in knowledge and behavior, comparable to teacher-led models.	Use peer-led strategies to complement teacher/dentist-led approaches, particularly in resource-limited settings.
Teacher-led education	Classroom-based lectures and demonstrations led by teachers integrated into existing school curricula.	Significant gains in knowledge and practical behaviors with consistent reinforcement over time.	Train teachers as oral health educators to ensure continuity in resource-limited areas with limited dental professional access.
Dentist-led education	Dental professionals delivering educational sessions with specialized knowledge and clinical focus.	Highest effectiveness in improving knowledge and behavior among all tested models.	Prioritize dentist involvement where resources allow, while leveraging other models to supplement access.
Referral programs	Combining education with referrals to dental clinics for preventive or curative care.	Increased dental service utilization, though socio-economic barriers persisted among low-income groups.	Integrate referral programs with subsidies or support systems to reduce access barriers for disadvantaged populations.
Gender-specific tailoring	Accounting for gender differences in responsiveness to oral health education.	Female students showed higher knowledge and behavior improvements post-intervention	Develop gender-sensitive interventions that cater to the unique learning and engagement styles of boys and girls.

A key observation in the reviewed studies is the geographic concentration. Most of the research has been conducted in Nigeria's South-East and South-West regions, specifically in cities like Enugu, badan, and Lagos. While these urban centers provide a valuable setting for studying school-based interventions, they do not reflect the diversity of the Nigerian population, particularly the largely rural and semi-urban areas that face even greater healthcare access challenges. In addition, the absence of studies from the northern and middle-belt regions leaves a gap in understanding how different socio-cultural dynamics may influence the effectiveness of oral health programs in these areas. The limited number of dental schools in the northern and middle-belt regions with few epidemiologists and public oral health specialists may have contributed to this observation. The ongoing efforts to strengthen the presence of dental schools in Northern Nigeria may result in changes in context specific knowledge generation in the near future.

## Conclusions

The current scoping review highlighted that school-based oral health interventions in Nigeria are focused on oral health education, with most of the studies aimed at improving oral hygiene practices. Oral health education was conducted through verbal communication and complemented with technology in a few cases. Peers, teachers and dentists were also assessed as oral health educators for school-based oral health interventions. However, the studies on school-based oral health interventions in Nigeria are few and skewed to southern Nigeria. Since the cultural and economic differences between northern and southern Nigeria can significantly influence the effectiveness of oral health interventions, there is the need to study Intervention in northern Nigeria.

## Data Availability

The original contributions presented in the study are included in the article/Supplementary Material, further inquiries can be directed to the corresponding author.
