# Supplementary material for: A scoping review of school-based oral health interventions among adolescents in Nigeria
Source: Front Oral Health. 2025 Jul 29;6:1577753. doi: 10.3389/froh.2025.1577753 (PMC12339554; doi:10.3389/froh.2025.1577753)
Supplement: Supplementary file 1 [file Table1.docx]

Supplementary Material

# Supplementary Tables

Appendix A

**Keywords and MeSH Terms used in PubMed Search:**

School Health Services

● Health Service, School

● School Health Service

● Service, School Health

● School-Based Services

● School Based Services

● School-Based Service

● Service, School-Based

● Services, School-Based

● Services, School Health

● School-Based Health Services

● Health Service, School-Based

● Health Services, School-Based

● School Based Health Services

● School-Based Health Service

● Service, School-Based Health

● Services, School-Based Health

● Health Services, School

● School Health Promotion

● Health Promotion, School

● Health Promotions, School

● Promotion, School Health

● Promotions, School Health

● School Health Promotions

Oral Health

● Dental Clinics

● Dental Health Surveys

● Diagnosis, Oral

● Mouth Diseases

● Mouth Rehabilitation

Adolescents

● Adolescents

● Adolescence

● Teens

● Teen

● Teenagers

● Teenager

● Youth

● Youths

● Adolescents, Female

● Adolescent, Female

● Female Adolescent

● Female Adolescents

● Adolescents, Male

● Adolescent, Male

● Male Adolescent

● Male Adolescents

Nigeria

● Federal Republic of Nigeria

**Search Strings**

PubMed:

("School Health Services" OR "School-Based Intervention") AND ("Oral Health" OR "Dental Health") AND ("Adolescents" OR "Teenagers") AND "Nigeria"

Scopus:

TITLE-ABS-KEY(("School Health Services" OR "School-Based Intervention") AND ("Oral Health" OR "Dental Health") AND ("Adolescents" OR "Teenagers") AND "Nigeria")

Web of Science:

TS=("School-Based" OR "School-Based Intervention") AND TS=("Oral Health" OR "Dental Health") AND TS=("Adolescents" OR "Teenagers") AND TS="Nigeria"

Google Scholar:

"School-Based" OR "School-Based Intervention" AND "Oral Health" OR "Dental Health" AND "Adolescents" OR "Teenagers" AND "Nigeria"

African Journals Online:

("School-Based" OR "School-Based Intervention") AND ("Oral Health" OR "Dental Health") AND ("Adolescents" OR "Teenagers") AND "Nigeria".
